# Epstein-Barr Virus-Positive Cancers Show Altered B-Cell Clonality

**DOI:** 10.1128/mSystems.00081-18

**Published:** 2018-09-25

**Authors:** Sara R. Selitsky, David Marron, Lisle E. Mose, Joel S. Parker, Dirk P. Dittmer

**Affiliations:** a Lineberger Comprehensive Cancer Center, School of Medicine at the University of North Carolina at Chapel Hill, Chapel Hill, North Carolina, USA; b Department of Genetics, University of North Carolina at Chapel Hill, Chapel Hill, North Carolina, USA; c Department of Microbiology and Immunology, University of North Carolina at Chapel Hill, Chapel Hill, North Carolina, USA; Harvard Medical School

**Keywords:** B cells, EBV, Epstein-Barr virus, TCGA, virology, cancer, herpesvirus

## Abstract

Epstein-Barr virus (EBV) is convincingly associated with gastric cancer, nasopharyngeal carcinoma, and certain lymphomas, but its role in other cancer types remains controversial. To test the hypothesis that there are additional cancer types with high prevalence of EBV, we determined EBV viral expression in all the Cancer Genome Atlas Project (TCGA) mRNA sequencing (mRNA-seq) samples (*n* = 10,396) from 32 different tumor types. We found that EBV was present in gastric adenocarcinoma and lymphoma, as expected, and was also present in >5% of samples in 10 additional tumor types. For most samples, EBV transcript levels were low, which suggests that EBV was likely present due to infected infiltrating B cells. In order to determine if there was a difference in the B-cell populations, we assembled B-cell receptors for each sample and found B-cell receptor abundance (*P* ≤ 1.4 × 10^−20^) and diversity (*P* ≤ 8.3 × 10^−27^) were significantly higher in EBV-positive samples. Moreover, diversity was independent of B-cell abundance, suggesting that the presence of EBV was associated with an increased and altered B-cell population.

**IMPORTANCE** Around 20% of human cancers are associated with viruses. Epstein-Barr virus (EBV) contributes to gastric cancer, nasopharyngeal carcinoma, and certain lymphomas, but its role in other cancer types remains controversial. We assessed the prevalence of EBV in RNA-seq from 32 tumor types in the Cancer Genome Atlas Project (TCGA) and found EBV to be present in >5% of samples in 12 tumor types. EBV infects epithelial cells and B cells and in B cells causes proliferation. We hypothesized that the low expression of EBV in most of the tumor types was due to infiltration of B cells into the tumor. The increase in B-cell abundance and diversity in subjects where EBV was detected in the tumors strengthens this hypothesis. Overall, we found that EBV was associated with an increased and altered immune response. This result is not evidence of causality, but a potential novel biomarker for tumor immune status.

## Abstract (precis)

Around 20% of human cancers are associated with viruses. Epstein-Barr virus (EBV) contributes to gastric cancer, nasopharyngeal carcinoma, and certain lymphomas, but its role in other cancer types remains controversial. We assessed the prevalence of EBV in RNA-seq from 32 tumor types in the Cancer Genome Atlas Project (TCGA) and found EBV to be present in >5% of samples in 12 tumor types. EBV infects epithelial cells and B cells and in B cells causes proliferation. We hypothesized that the low expression of EBV in most of the tumor types was due to infiltration of B cells into the tumor. The increase in B-cell abundance and diversity in subjects where EBV was detected in the tumors strengthens this hypothesis. Overall, we found that EBV was associated with an increased and altered immune response. This result is not evidence of causality, but a potential novel biomarker for tumor immune status.

## INTRODUCTION

Approximately 20% of human cancers are associated with infectious agents (1), many with DNA tumor viruses. Simply finding DNA sequences or evidence of viral transcription in a given tumor does not necessarily mean that the virus contributes mechanistically to tumorigenesis. That determination requires fulfilling Koch’s postulates or their modern, genomic-based equivalents (2). Examples of overinterpreted reports abound, many due to laboratory contaminations (3), as in the case of simian virus 40 (SV40) (4), xenotropic murine leukemia virus-related virus (5), or the finding of HeLa cell-resident human papillomavirus type 18 in non-cervical cancer Cancer Genome Atlas Project (TCGA) mRNA sequencing (mRNA-seq) data (6, 7). This led to very stringent criteria, such as requiring at minimum 1,000 bp of combined genome coverage or >1,000 individual reads/sample. We hypothesized that the tumor represents a specialized environment within the body, which may selectively attract, retain, and/or foster localized replication of viruses and of virus-infected cells, even if these viruses did not have a causal role in tumor development. These would be present at low levels. Accordingly, it is important to view the results presented here not as evidence for causality, but as potentially novel biomarkers with clinical implications.

Epstein-Barr virus (EBV) is a human herpesvirus, and like the other seven human herpesviruses, EBV establishes lifelong persistence in the human host, a state that is termed latency. Over 90% of adults are infected with EBV by age 50, and at any given time, a third of seropositive persons shed infectious virus in saliva (8). Once infected, the EBV genome is continuously present in circulating CD38-positive memory B cells (9). During this state, no or only a very few viral mRNAs are transcribed. Spontaneous mononucleosis or immunodeficiency-associated reactivation events result in amplification of EBV-infected cells and extended mRNA transcription. *De novo* B-cell and epithelial cell infection are associated with widespread, promiscuous transcription across the entire ∼172,000-bp viral genome. Unlike the other human herpesviruses, except for human herpesvirus 8 (Kaposi sarcoma-associated herpesvirus), EBV is strongly associated with cancer and able to transform primary human B cells in culture. There exists sufficient evidence to associate EBV infection with Burkitt lymphoma (BL), Hodgkin's disease (HD), posttransplant lymphoproliferative disease (PTLD), and rare forms of non-Hodgkin's lymphoma, such as those localized to the central nervous system in AIDS patients (AIDS-CNS lymphoma) (1). These cancers originate from B cells, and the virus is present at multiple copies in every tumor cell. The exception is HD, where only a fraction of the proliferative compartment, the Reed-Sternberg cell, is EBV positive. HD is one example of a cancer that is convincingly and causally virus associated, yet the virus is present in only a fraction of the cells in the lesion. In addition, EBV is associated with the epithelial cancers nasopharyngeal carcinoma (NPC) and gastric cancer. Here again, the EBV genome and EBV mRNAs are found in every single tumor cell. Because cancers are a heterogeneous group of diseases, the EBV association ranges from >90% in AIDS-CNS lymphoma to approximately 10% in gastric cancer (10). Furthermore, many associations depend on geographic locale. For instance, most cases of EBV-positive NPC are observed in southern China and among the Inuit in the Arctic (11).

In addition to these bona fide associations, which are verified by multiple, independent epidemiological studies, EBV DNA, protein, and RNAs have been found in the biopsy material of many other cancers. In the case of breast cancers, discordant results “for” and “against” the presence of EBV in a given tumor have been published (12, 13). In these cases, it is unclear if (i) EBV contributes mechanistically to tumor development, (ii) is a “passenger” virus due to the presence of infected, infiltrating B cells, or (iii) represents isolated instances of local abortive infection. To more broadly explore the association between EBV and cancers of different lineages, we queried the entire TCGA mRNA-seq data set, representing 10,396 cancer cases, for the presence of EBV transcripts.

## RESULTS

### Epstein-Barr virus is present in many tumor types.

To detect viruses from RNA-seq data, we used VirDetect, software based on the principles of digital subtraction (14). We deployed the algorithm on the Google Compute platform. We developed VirDetect for two reasons: (i) we could not find public work flows for detection of many viruses at once, and (ii) many of the published work flows require manual curation after alignment due to poor specificity. The poor specificity is caused by the areas of low complexity and human homology found in some viral genomes. The added value of VirDetect is the masked genome, which leads to very few false-positive alignments. To test VirDetect’s ability to detect selected viruses, we simulated random reads from EBV and four other viruses from different genomic classes (see Fig. S1 in the supplemental material). We estimated the false-positive rate as 0% and the false-negative rate as 8% for EBV: i.e., 8% of reads drawn from EBV did not realign back to an EBV genome as masked by VirDetect. These represent repeat and/or low-complexity regions that are present in the virus.

10.1128/mSystems.00081-18.1 FIG S1 Efficiency of mapping. The vertical axis represents the percentage of simulated virus reads drawn from each virus that aligned back to the correct virus. One hundred samples of 1,000 random paired-end reads for each virus were simulated. Boxes represent median ± interquartile range and whiskers median ± 1.5× interquartile range. Outliers are represented by black dots. The horizontal categories represent dengue virus 3 (DENV3), which has ∼10,000 nucleotides (nt) and is a positive-strand RNA virus, Epstein-Barr virus (EBV), which has ∼172,000 nt and is a double-stranded DNA virus ubiquitous in the human population and coevolved since speciation, human herpesvirus 7 (HHV7), which has ∼145,000 nt and is a double-stranded DNA virus ubiquitous in the human population with known regions of sequence similarity to human sequences and coevolved since speciation, human immunodeficiency virus 1 (HIV1), which is an ∼9,200-nt retrovirus and was recently introduced into the human population, and Ebola virus (Ebola), which has ∼19,000 nt and is a negative-strand RNA virus, causing sporadic outbreaks. It is not stable in the human population. Download FIG S1, TIF file, 0.1 MB. Copyright © 2018 Selitsky et al. 2018 Selitsky et al. This content is distributed under the terms of the Creative Commons Attribution 4.0 International license.

**FIG 1:**
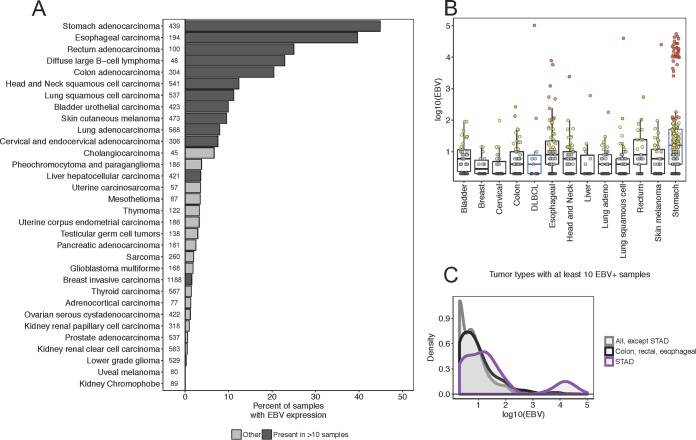
EBV prevalence and abundance in 10,396 (*n*) human tumors. (A) Bar plot of the percentage of samples with EBV mRNA (at least one paired-end read that aligned to the EBV genome) for all TCGA tumor types. Dark gray indictates that tumor type had at least 10 samples with EBV mRNA; light gray indicates there were <10 samples. The number before each bar is the number of samples in each cohort. (B) EBV expression (log_10_ [raw EBV counts]) for tumor types with at least 10 subjects with EBV expression. Boxes represent median ± interquartile range and whiskers median ± 1.5× interquartile range. The color of the box represents if the tumor type was determined by WHO to be associated with EBV (blue) or not (black). The color of the dot represents EBV transcript level (gray, <10; yellow, 10 to 100; orange, 100 to 1,000; red, >1,000. (C) Density distribution for all tumor types except gastric cancer (STAD [gray]), colon, rectal, and esophageal combined (black), and STAD (purple). Note the pronounced bimodal distribution of EBV read count for STAD.

We used VirDetect to query all mRNA-seq samples from the TCGA (*n* = 10,396 samples, 32 tumor types) for evidence of transcripts originating from any of 1,894 vertebrate viruses. EBV was present in at least some samples among most tumor types, and present in >10 samples in 13 tumor types (Fig. 1A). We required a minimum of 10 samples per tumor type for exploratory association analyses; further analyses focused only on these tumor types. The EBV mRNA-positive (EBV^+^) tumor types included stomach cancer (STAD) and diffuse large B-cell lymphoma (DLBCL), as expected. The remaining tumor types were breast, bladder, cervical, colon, esophageal, head and neck, liver, lung (both adenocarcinoma and squamous cell carcinoma), rectal, and skin cutaneous melanoma. As a positive control, we used human endogenous retrovirus transcription (HERV-K group) mRNA, which was detected in every sample (15).

EBV was present in only a few DLBCL samples, as the TCGA data set is comprised largely of sporadic DLBCL and contains very few pediatric Burkitt, CNS, or HIV-associated DLBCLs. In one striking case, EBV-mapped read counts peaked at over 100,000 reads, which amounted to 0.5% of all mapped reads for this sample (Fig. 1B). EBV was detected in 45% of STAD samples. The expression of EBV in STAD was bimodal, with two populations (a) ranging from 2 to 200 and (b) 1,000 to 100,000 counts (Fig. 1B and C). STAD samples with the higher range of EBV expression were independently determined as belonging to the EBV molecular subtype (see Fig. S2 in the supplemental material) (16). For these, we expect that EBV was present in the epithelial lineage tumor cell itself. EBV expression in the lower range, which was predominant among all other positive cancer types, may be due to infiltrating B cells, as previously described by Ryan et al. (10) for gastric adenocarcinoma samples.

10.1128/mSystems.00081-18.2 FIG S2 Validation of thresholds for establishing the presence of EBV and demonstration of consistency with prior classifications in the TCGA. Shown is a stacked bar plot of EBV expression for each gastric cancer molecular subtype defined by the TCGA. Bars are colored by EBV sequence read abundance: absent (gray), present with low counts (<1,000 raw counts [yellow]), or present with high counts (>1,000 raw counts [red]). The vertical axis indicates the number of samples in each category (*n* = 295). The TCGA network defined EBV-positive gastric cancers as >1,000 bp (noncontiguous) of pathogen recovered. Specifically, 75-nt mRNA reads were aligned using a filtering algorithm that required 2/3 to align to EBV. Counts were normalized to EBV genome size × 10^6^/chastity passed reads. On average, the normalized EBV counts for the 24 (10%) EBV-positive tumors have 206-fold greater normalized reads than the EBV-negative tumors. The cutoff used here recovered all TCGA EBV-positive tumors, which clearly represent the cases with the highest number of EBV sequence reads, but also identified bona fide EBV transcripts in a substantial fraction of the other molecular subtypes. Download FIG S2, TIF file, 0.1 MB. Copyright © 2018 Selitsky et al. 2018 Selitsky et al. This content is distributed under the terms of the Creative Commons Attribution 4.0 International license.

Esophageal carcinoma had the second highest percentage of samples with EBV mRNAs (40%), which may be due to some of the samples being in anatomical proximity to the stomach and occasionally indistinguishable from gastric cancer samples (17, 18). The esophageal carcinoma samples collected from the junction between the esophagus and the stomach were significantly enriched for EBV^+^ samples compared to samples from other esophageal anatomical sites (chi-square test, *P*  ≤ 0.0004 [see Fig. S3 in the supplemental material]) and were among the “high” read class (red in Fig. 1B). EBV mRNA was also present in colon and rectal cancers at elevated frequencies, but with low read counts. These broad tumor types—stomach, esophageal, colon, and rectal—have a commonality in that each was from a digestive gastrointestinal organ.

10.1128/mSystems.00081-18.3 FIG S3 Distribution of EBV in gastric cancer by anatomical location. Shown are the total number of cases (upper panels) and the proportion of samples (lower panels). Bar plots of the esophageal and gastric cancer tumor’s anatomical location, as reported by TCGA, are split by tumor type—gastric (STAD) and esophageal carcinoma (ESCA). Bars are colored by EBV sequence read abundance: absent (gray), present with low counts (<1,000 raw counts [yellow]), or present with high counts (>1,000 raw counts [red]). The vertical axis indicates the number of samples in each category (*n* = 164). Download FIG S3, TIF file, 0.3 MB. Copyright © 2018 Selitsky et al. 2018 Selitsky et al. This content is distributed under the terms of the Creative Commons Attribution 4.0 International license.

A number of TCGA samples had adjacent nontumor material available from the same patient. Nine of 13 tumor types had >10 samples with matched adjacent tissue. Among this set of matched samples, EBV expression was enriched in the tumor overall (chi-square test, *P*  ≤ 6.6 × 10^−8^) and increased individually in most tumor types (see Fig. S4 in the supplemental material). Observing an enrichment in tumor tissue compared to nontumor tissue is consistent with the hypotheses that EBV (i) was present in the tumor cell itself or (ii) was present due to infiltrating B cells. Observing an enrichment is inconsistent with the hypothesis of random sample contamination. B cells should not be present at large amounts in nonlymphatic and noncancerous tissue, unless there is an infection, but B cells may be abundant in tumors due to increased angiogenesis or the immune response to the tumor.

10.1128/mSystems.00081-18.4 FIG S4 Number of tumor samples that were positive for EBV compared to nontumor-adjacent controls. The proportions of samples are shown on the upper panel, and the raw numbers of samples are shown in the lower panel. Each facet is separated by the sample of origin, comparing cancer tissue to adjacent noncancer tissue. Only cancer types with ≥10 noncancer-adjacent samples are included. Color represents EBV status. TCGA pathology review criteria stipulate >60% tumor nuclei and <20% necrosis in tumor specimens and no evidence of tumor cells in adjacent noncancer tissue. Note how in esophageal carcinoma, EBV is essentially absent from adjacent noncancer tissue, while in gastric cancer (stomach), EBV is present in an almost equal portion of cancer and adjacent noncancer tissues. Download FIG S4, TIF file, 0.3 MB. Copyright © 2018 Selitsky et al. 2018 Selitsky et al. This content is distributed under the terms of the Creative Commons Attribution 4.0 International license.

### Epstein-Barr virus is associated with B-cell signatures.

**FIG 2:**
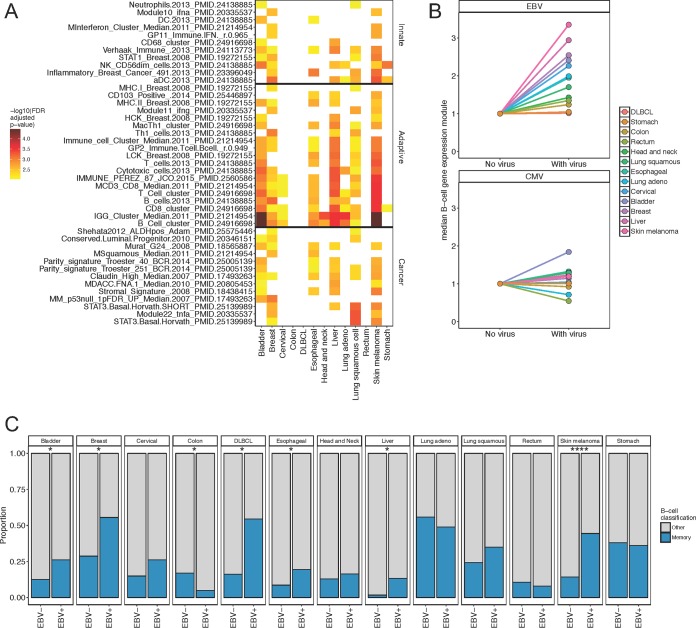
EBV presence is associated with altered gene expression. (A, B, and C) Tumor types with >10 EBV^+^ samples are included. (A) Differential expression of gene expression module heat map for samples with and without expression of EBV. The color of the tile represents –log_10_ (FDR-adjusted *P* value) determined by Mann-Whitney *U* test. If the *P* value is <0.01, then it is colored from yellow to red to black: otherwise the tile is white. The modules included have at least two cancer types with a corrected *Q* value of <0.01. (B) Median expression of B-cell gene expression module for each tumor type, separated by expression status (“With virus”) or no expression (“No virus”) of virus (either EBV or CMV), normalized to 1 for no virus. Each tumor type is represented by a different color. The top panel shows results for EBV, and the bottom panel shows results for CMV. (C) Stacked proportional bar plot separated by EBV expression (EBV^+^) or no EBV expression (EBV^−^) for each tumor type. Color represents the classification for BAGS (see methods), either memory or other B-cell types. Asterisks indicate nominal significant enrichment of a classification determined using chi-square test: *, *P*  < 0.05; ****, *P* < 0.001.

To formally test the hypothesis that EBV positivity was associated with a specific tumor resident or infiltrating cell type, we tested if any gene expression module (a group of correlated genes, summarized by a single value [19]) was differentially expressed between EBV^+^ and EBV^−^ samples. Eighty out of 485 gene expression modules tested were significantly differentially expressed (Mann-Whitney *U* test, *q* <  0.01, after false-discovery rate [FDR] adjustment by the Benjamini-Hochberg procedure) in at least one tumor type and 43 in two or more tumor types (Fig. 2A). Most of the modules were immune related, such as natural killer (NK) and B- and T-cell modules (all upregulated in EBV^+^ samples), consistent with the hypothesis that infiltrating immune cells are the carrier of the EBV signal. Other modules were tumor or inflammation related, such as P53 and STAT3. In 9/13 (69%) tumor types, B-cell-associated modules were significantly differentially expressed (maximum *q* value of <0.01 across these tumor types).

The B-cell gene expression module had significantly higher median expression in EBV^+^ samples compared to EBV^−^ samples in 11/13 (84%) tumor types (linear regression conditioning on tumor type, *P*  < 2 × 10^−16^) (Fig. 2B). As expected B-cell gene expression was not increased in EBV^+^ stomach cancer, as the virus resides in epithelial cells (Fig. 2B, orange). Also, as expected, DLBCL did not show an increase in B-cell gene expression, since by definition all DLBCLs are of a B-cell lineage. As a control, we evaluated the association between B-cell gene expression and human cytomegalovirus (HCMV/HHV5), a ubiquitous herpesvirus of comparable genome size. There was no significant association with the presence of this virus and B-cell gene expression, except in bladder cancer (unadjusted *P* value of ≤0.02 by Mann-Whitney *U* test, FDR-adjusted *q* value of ≤0.17) (Fig. 2B). The association between the B-cell gene expression and HCMV in bladder cancer is consistent with the biology of HCMV. Whereas there is not enough evidence to establish a direct connection between HCMV and bladder cancer, one would expect to find HCMV in bladder tissue, as this herpesvirus, unlike EBV, is transmitted to a large degree by urine and shed continuously throughout life (20). Among the tumor types, melanoma and liver cancer had the greatest increase of the B-cell gene expression module in EBV^+^ samples compared to EBV^−^ samples.

### Memory B-cell phenotype is enriched in tumor samples with Epstein-Barr virus.

EBV establishes latency in memory B cells, and at any given time, a percentage of EBV-positive CD20^+^ IgD^−^ CD38^+^ memory B cells are circulating in the blood (9). In contrast, active EBV replication/reactivation induces a B-cell blast phenotype and results in the *de novo* infection of naive B and epithelial cells. To test the hypothesis that memory B cells were enriched in EBV^+^ tumors, we used machine learning to build a classifier from previously published transcription profiles of sorted human B cells (GSE56315) (21). The B-cell transcriptional profiles were divided into memory, centroblast, centrocyte, plasmablast, and naive B-cell types. We classified all the tumor samples according to which cell type had the greatest similarity by expression using distance-weighted discrimination (22, 23). Using a generalized linear model and adjusting for tumor type, we found that the assigned memory phenotype probability was significantly higher (*P*  ≤ 4.1 × 10^−7^), while the naive, nonactivated, phenotype was significantly lower in the EBV^+^ samples (*P*  ≤ 1.1 × 10^−12^) (Table 1). Assessing each of the 13 tumor types individually, we found that EBV-positive samples were enriched in the memory B-cell phenotype in 6/13 (46%) tumor types by chi-square test (*P* ≤ 0.05) (Fig. 2C). This observation is consistent with the hypothesis that the tumor-associated EBV signal stems from tumor-infiltrating, perhaps activated, memory B cells rather than naive B cells.

**TABLE 1:** EBV presence is associated with different B-cell phenotypes

Phenotype^*a*^	*P* value	Effect size
Centroblast	0.05	−0.43 (−0.85 to 0.00)
Centrocyte	0.02	0.24 (0.04 to 0.45)
Memory	4.1 × 10^−7^	0.70 (0.42 to 0.96)
Naïve	1.1 × 10^−12^	−0.59 (−0.75 to −0.43)
Plasmablast	0.14	−0.09 (−0.22 to 0.03)

a Shown are the *P* value and effect size from a generalized model with EBV status and cancer type as the predictor and logit transformation of the B-cell phenotype probabilities as the outcome.

### Epstein-Barr virus is associated with increased B-cell diversity.

**FIG 3:**
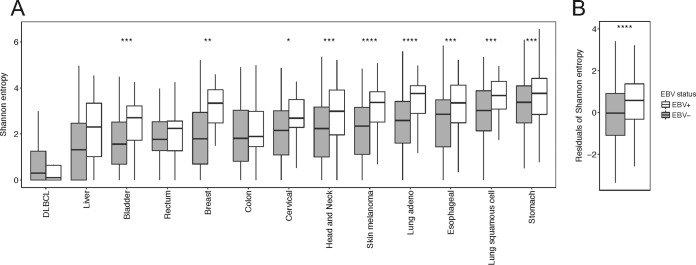
EBV^+^ samples have increased B-cell receptor diversity. (A and B) Boxes represent median ± interquartile range and whiskers median ± 1.5× interquartile range. White indicates EBV-positive and gray EBV-negative samples. The tumor types included have EBV expression in >10 subjects. (A) Box plots displaying Shannon entropy. (B) Plotted residuals from a generalized linear model with cancer types as the predictor and Shannon entropy as the outcome. *, *P*  < 0.05; **, *P*  < 0.01; ***, *P*  < 0.001; ****, *P* < 0.001. Outliers are not present.

EBV infection is known to induce cell proliferation and expansion of the infected B cells. Hence, one would expect that a reactivation or *de novo* infection event would lead to a change in B-cell diversity. To assess B-cell diversity in EBV^+^ tumor samples, we assembled B-cell receptors (BCRs) using V’DJer (24) and calculated diversity using Shannon entropy. The discussed BCRs are from heavy-chain IgG, which is the chain associated with an activated B-cell response (naive B cells bear IgD or IgM isotypes). Using linear regression, conditioning on the 13 tumor types with more than 10 EBV samples, we found that samples with EBV had (i) significantly increased total BCR counts (*P* ≤ 1.4 × 10^−20^) and (ii) higher BCR diversity (*P* ≤ 8.3 × 10^−27^). We also tested each cancer type individually, using the Mann-Whitney *U* test. As expected, DLBCL, which is a monoclonal cancer of B cells, exhibited the lowest diversity (median = 0.29) and had no difference between samples by EBV status (Fig. 3A). In contrast, stomach cancer exhibited the highest diversity and had a significant enrichment of diversity in EBV^+^ samples (*P*  < 0.001) (Fig. 3A). Most tumor types had higher BCR abundance as well as BCR diversity in the EBV^+^ samples.

Shannon entropy is a composite measure of both species evenness and abundance. To determine if Shannon entropy was only higher due to its dependence on abundance, or whether there was truly a diversity difference in samples with EBV expression, we tested a model conditioning on all 13 cancer types together as well as counts and found that diversity was still significant after conditioning on counts (*P*  ≤ 3.61 × 10^−14^ by linear regression) (Fig. 3B). In particular, melanoma, lung adenocarcinoma, head and neck cancers, and bladder urothelial carcinoma were significantly associated with diversity after first conditioning on counts (*P*  ≤ 0.01, 0.004, 0.009, and 0.009, respectively).

We assessed if the presence of any other virus was associated with BCR abundance and diversity and found that only EBV was associated with these two parameters (see Fig. S5 in the supplemental material); however, as most of the other viruses were not as prevalent as EBV, we had less power to detect such an association. These data are consistent with enrichment of class-switched, polyclonal B cells in EBV^+^ tumor samples.

10.1128/mSystems.00081-18.5 FIG S5 Differential expression of B-cell receptor measurements comparing samples with and without expression for EBV strains I and II and other viruses in the VirDetect database. First, two measures, total counts and Shannon entropy, for each sample were obtained for assembled IgG and IgA heavy chains (IGHG and IGHA, respectively). Second, the values for virus-positive to virus-negative samples were compared by Mann-Whitney *U* test. Tiles were only included if the adjusted *P* value was <0.05, as determined using Mann-Whitney *U* test: otherwise the tile is absent. The tile colors are yellow to red to black, representing –log_10_ (*q* value), the Benjamini-Hochberg-corrected *P* value. Vertical black dividing lines separate the IGHG and IGHA measurements. Naturally, FDR-adjusted *P* values are higher for cancers with a higher proportion of EBV-positive cases (e.g., stomach and esophageal). In contrast to EBV, HCMV did not show differential B-cell receptor measurements between virus-positive and virus-negative cases. In the case of EBV, two type-specific reference strains were used. The first was NC_007605.1 (Epstein-Barr virus type 1), which was assembled from data for the B95-8 (V01555) and Raji (M35547) strains with the following corrections. The number of IR1 (W) repeats in V01555 has been reduced from 11.6 to a more typical 7.6, and the missing B95-8 sequence has been restored to give a sequence more representative of wild-type EBV. The second was NC_009334.1 (Epstein-Barr virus type 2), which was assembled from strain AG876. The data were too limited to associate one or the other type with specific cancers. Download FIG S5, TIF file, 0.3 MB. Copyright © 2018 Selitsky et al. 2018 Selitsky et al. This content is distributed under the terms of the Creative Commons Attribution 4.0 International license.

## DISCUSSION

We affirmed an association of EBV with DLBCL and a subset of gastric cancer (18, 25). In the case of esophageal carcinoma, the detection rate of EBV was associated with proximity to the stomach. As recently reported, it is difficult to distinguish some esophageal from gastric tumors (16, 17). EBV is ubiquitous in the adult population (26). EBV genomes are detectable in 0.004 to 0.01% of mononuclear cells in a healthy human (9). It is thus not surprising that we detected low-level EBV transcription in many samples. This likely stems from infiltrating B cells that carry EBV, as we observed an association between the overall B-cell expression signature and the presence of EBV.

In addition to EBV, other herpesviruses are also ubiquitous in the human population, such as HCMV/HHV5. We observed evidence of HHV5 transcription in a number of samples. Yet, HHV5 was not associated with B-cell gene expression, consistent with HHV5 establishing latency in myeloid progenitors and replicating in endothelial cells, but not B cells (27, 28).

In several tumor types, B-cell diversity was significantly increased even after first conditioning on B-cell content. This observation argues against selective retention or local expansion of a singular, EBV-infected clone. In a few tumor types, either EBV positivity alone or added as an interacting factor was associated with survival; however, we had limited confidence in these results due to the heterogeneity of how TCGA clinical data were collected and the heterogeneity of treatment of the cancer within a cohort. Nevertheless, it is easy and economical to test for EBV-specific transcripts in tumor biopsy specimens. Such a test may have utility as a predictive or prognostic biomarker. Examples for its use can be found in posttransplant lymphoproliferative disease, other types of EBV^+^ lymphoma (29–31), and NPC (32).

This study is an attempt to use systems biology to relate viral infections to immune responses as defined by transcript patterns, cell-type signatures, BCR diversity, and histopathology, such as inflammation and cancer immunotherapy. Immune signatures have been heralded as an assumption-free method of diagnosis driven by advances in machine learning, including many studies based on BCR diversity (33).The current data set was extremely heterogenous. This study could not decide whether EBV was first and inflammation and inflammatory cell (B, T, and NK) influx were second, or whether EBV-infected B cells were the result of tumor-associated inflammation, which attracted EBV-positive as well as EBV-negative B cells and led to EBV reactivation. Those insights are more likely to emerge in studies on specific tumors and populations: e.g., upper gastric carcinomas or response to immune modulatory therapy (34).

This study analyzed one of the most comprehensive mRNA-seq data sets to date to query the status of human viruses in cancer, both in terms of tumor diversity and total number of samples and reads. Prior studies had looked at the presence or absence of viruses in subsets of the TCGA (35–38) or across the general population (39) and thus had less power to detect possible virus-tumor associations. We used a variant of digital subtraction on RNA-seq data (14), thus requiring that viral mRNAs or long noncoding RNAs (lncRNAs) were expressed. This data set does not capture microRNAs. Digital subtraction and variations thereof represent the most widely used approach to “hunt” for viruses in sequencing data (40, 41) and have been credited with the detection of Merkel cell polyomavirus, among others (42). It has been validated for the detection of EBV in cell lines and other samples (39, 43, 44). We improved on the previous iterations of digital subtraction by first masking areas of low complexity and human homology. This masking step led to almost complete specificity. Hence, a less conservative threshold could be applied to identify EBV-positive samples than before. While this increased sensitivity is not necessarily supportive of establishing virus-tumor associations, it allows, for the first time exploration of an association between the presence of a virus (only EBV was found) and immune signatures of tumor-infiltrating cells.

As these were existing RNA-seq data, rather than biological specimens, we were not able to enrich for viral sequences by biochemical means prior to sequencing as in other studies using local patient cohorts (45–47). Thus, the sequencing depth of the original sample and the level of viral mRNAs limit this study’s sensitivity. In the context of cancer biology, this could be considered a strength and measure of specificity, as one would expect human oncoviruses to transcribe copious amounts of at least one of their oncogenes or oncogenic RNAs.

Because of the biology of human tumor viruses, this approach differs from broad metagenomic surveys (48)—e.g., of environmental samples or of diseases caused by acutely replicating viruses. Metagenomics is the description of all genome sequences within a sample and based on the assumption that multiple species coexist and all have some bearing on the necessarily composite phenotype. In contrast, of all viral tumors described to date, only a single virus—in fact a single strain or clonal integration event—is found in high abundance in the tumor biopsy specimen, and that virus transcribes a defined set of viral oncogenes, may they be protein-coding or noncoding RNAs. (The exception here is cancers caused by retroviral integration, where the virus induced the aberrant expression of human, but not viral, oncogenes [49].) EBV may be the one human virus most useful to evaluate with regard to tumor immune status.

## MATERIALS AND METHODS

### Virus detection, VirDetect.

VirDetect is available at https://github.com/dmarron/virdetect. RNA-seq reads were aligned to hg38 (without chrEBV) using STAR v2.4.2a (1,080 multi-maps, 10 mismatches). Unmapped reads were next aligned to a masked viral FASTA using STAR v2.4.2a (52 multi-maps, 5 mismatches). Vertebrate viral FASTA (1,894 viruses) was downloaded from GenBank. Viral FASTA was masked for increased specificity. Regions were masked in two ways. (i) Viral reads of length 75 were simulated from the entire viral FASTA and then mapped to hg38 using STAR v2.4.2a (1,080 multi-maps, 5 mismatches). If the virus simulated reads mapped to the human genome, they were masked in the viral FASTA. (ii) Areas of low complexity (9 or more repeating single nucleotides, 7 or more repeating double nucleotides, 4 or more repeating nucleotide patterns of 3, 3 or more repeating nucleotide patterns of 4, 2 or more repeating patterns of 5, or 2 or more repeating nucleotide patterns of 5) were masked. Viruses were then quantified using the resultant SAM file.

### *In silico* simulations.

Simulations were created using in-house scripts. A simulated sample was comprised of 1,000 50-bp paired-end reads randomly chosen (with replacement) from the genomes of 5 different viruses: NC_002549.1 (Ebola), NC_001802.1 (HIV-1), NC_001475.2 (dengue virus 3), NC_007605.1 (EBV), and NC_001716.2 (herpesvirus 7). For each virus, 100 samples were simulated.

### Computing gene expression modules.

Gene expression modules are groups of highly correlated genes together in a “module” (50). Each module was computed by Z-scaling each of the genes and then taking the median of value of the scaled genes.

### Assembly of B-cell receptors.

B-cell receptor repertoires were assembled for the immunoglobulin heavy chain across all TCGA mRNA-seq samples using V’DJer as described by Mose et al. (24).

### B-cell-type classification.

BAGS classifier (GSE56315) was built using linear distance-weighted discrimination (dwdLinear from the R package *Caret*) of genes with a standard deviation of log_2_-transformed RNA-seq of >0.2. The classification subtype of each sample was called by the subclassification with the highest probability.

### Statistical analysis.

All statistical analyses and plots were generated using R. Kaplan-Meier plots and Cox proportional hazards regression models were implemented using the R package *survival*. The R packages used for analyses were *stats*, *plyr*, *reshape2*, *doMC*, and *caret*. The R packages used for generating plots are *ggplot2* and *survival*.
