# Supplementary figures and images for: Epstein-Barr Virus-Positive Cancers Show Altered B-Cell Clonality
Source: mSystems. 2018 Sep 25;3(5):e00081-18. doi: 10.1128/mSystems.00081-18 (PMC6156273; doi:10.1128/mSystems.00081-18)

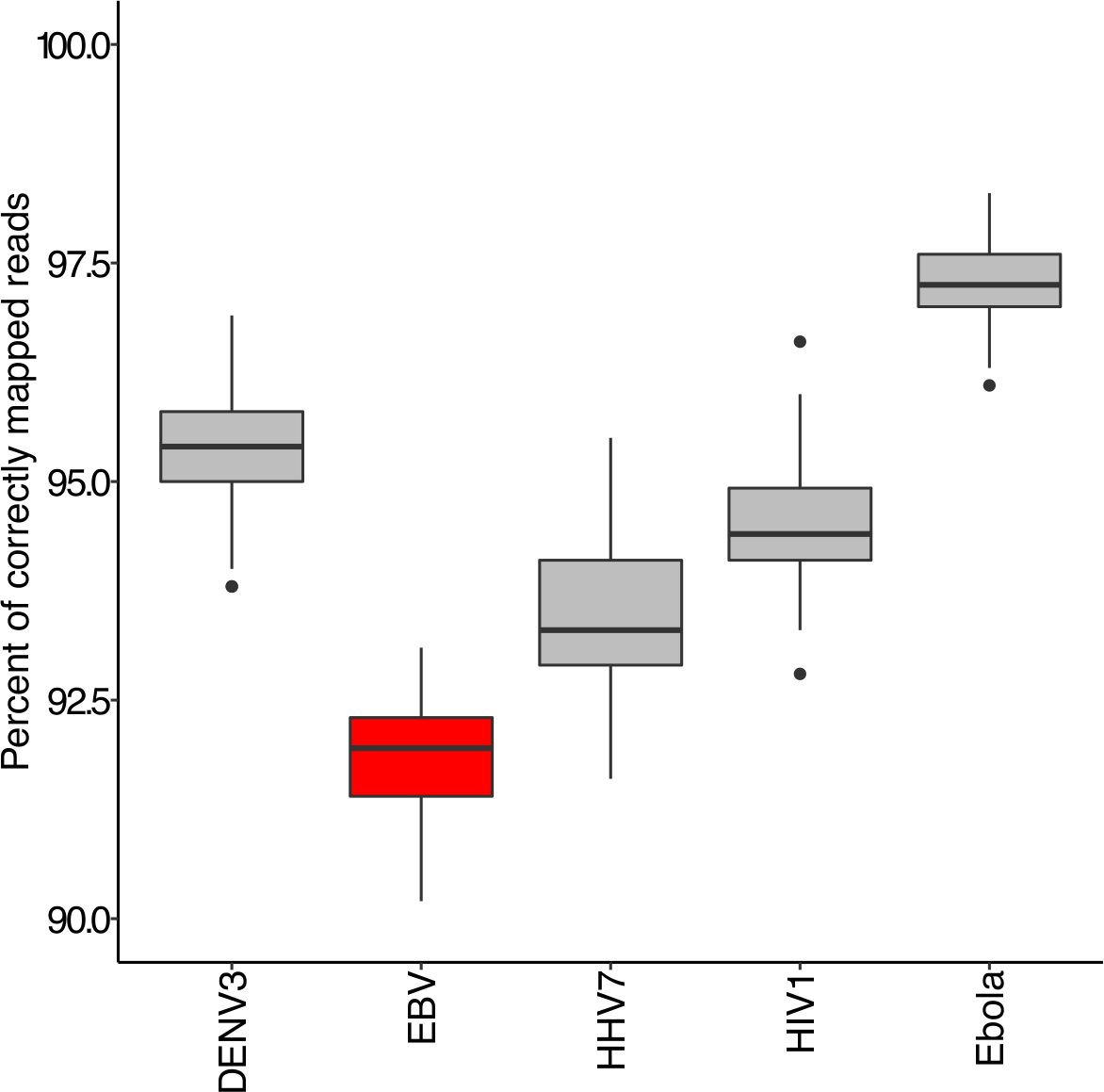

Supplement: FIG S1 [file sys005182263sf1.tif]

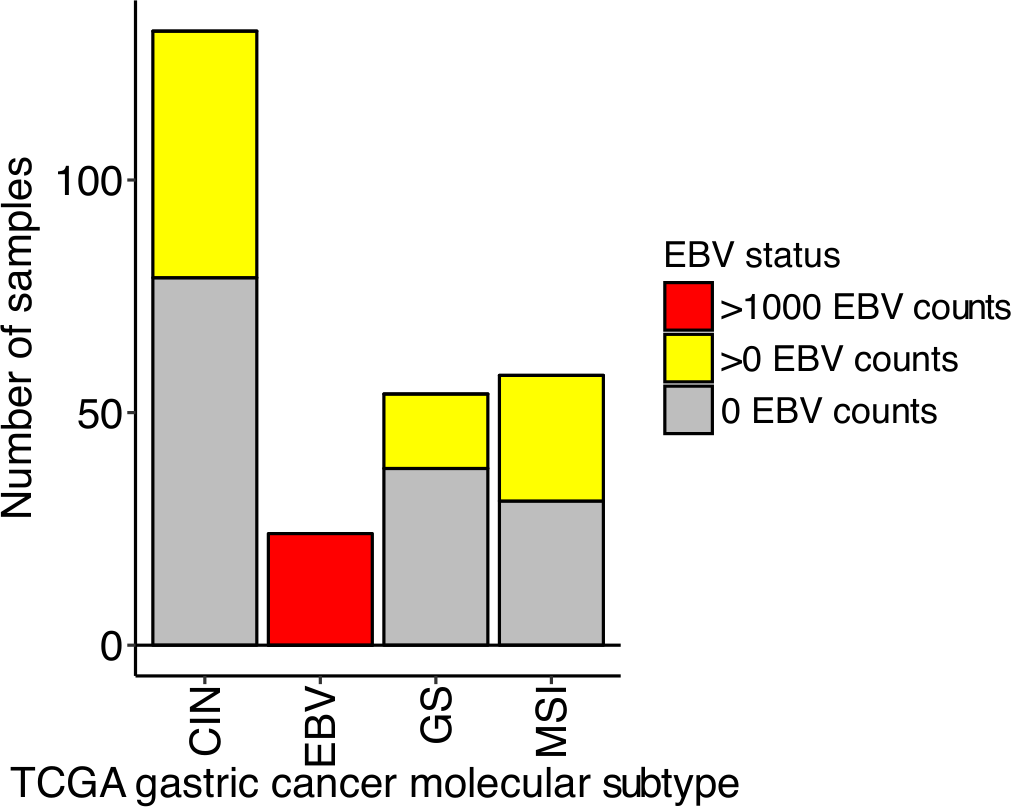

Supplement: FIG S2 [file sys005182263sf2.tif]

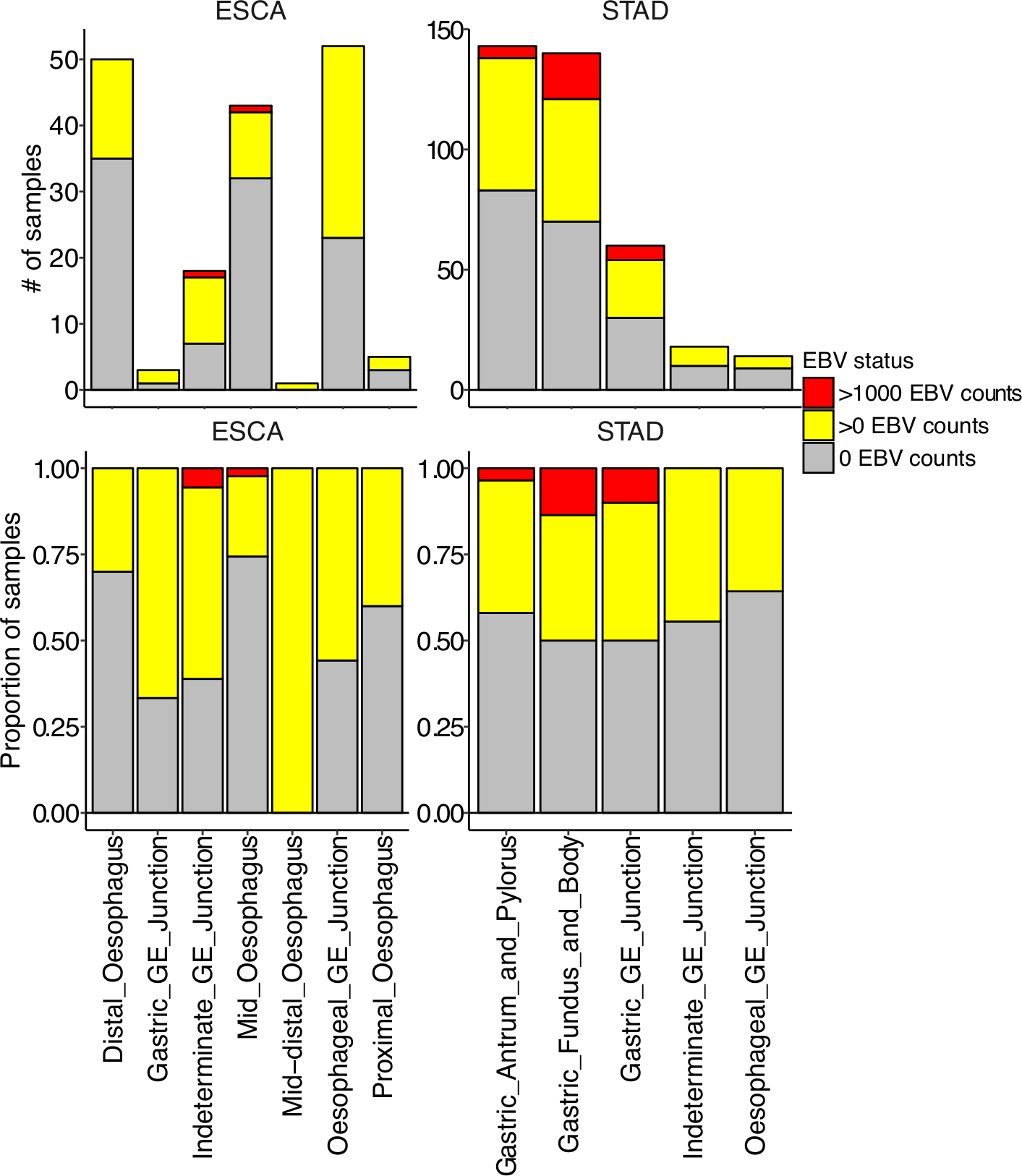

Supplement: FIG S3 [file sys005182263sf3.tif]

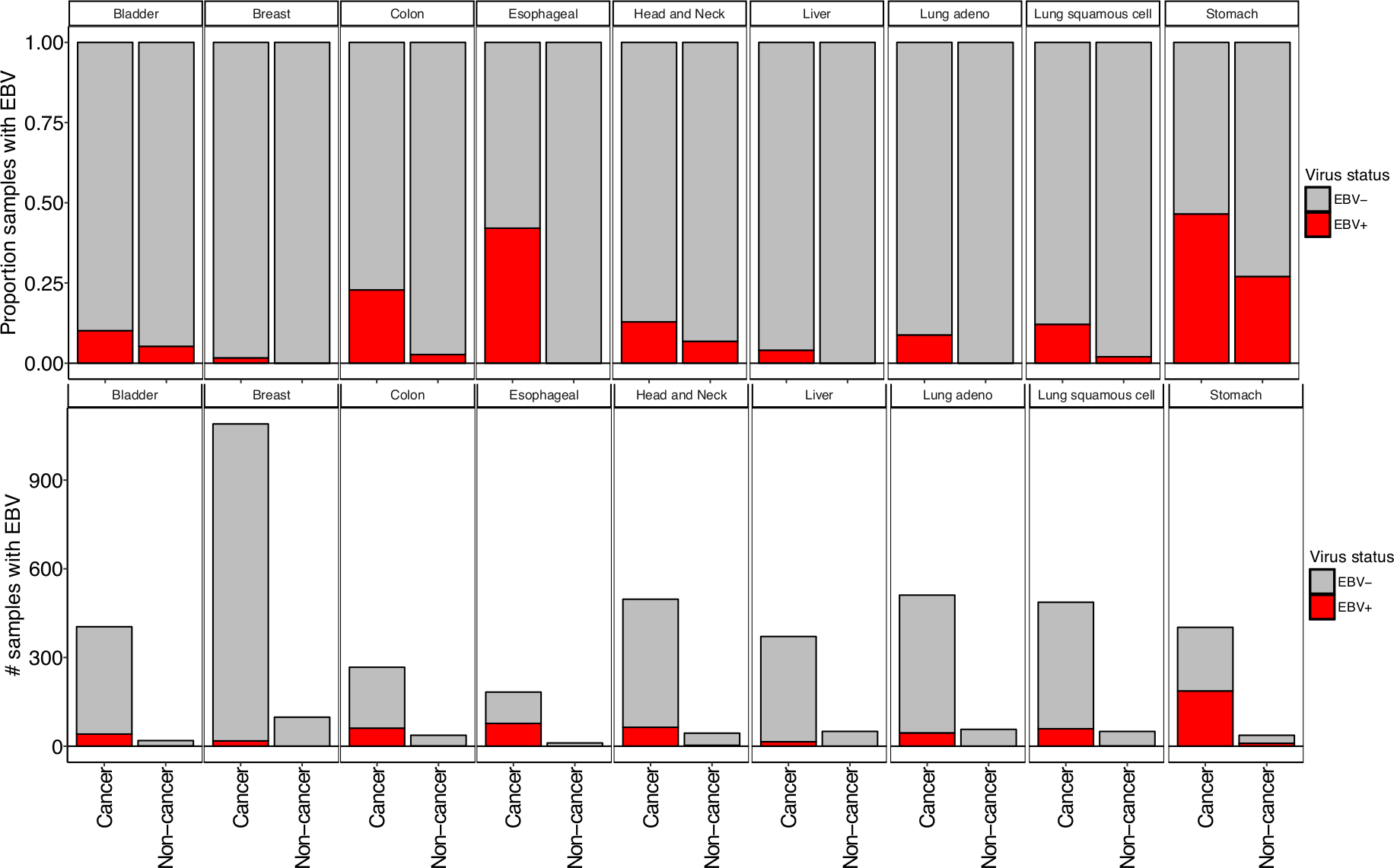

Supplement: FIG S4 [file sys005182263sf4.tif]

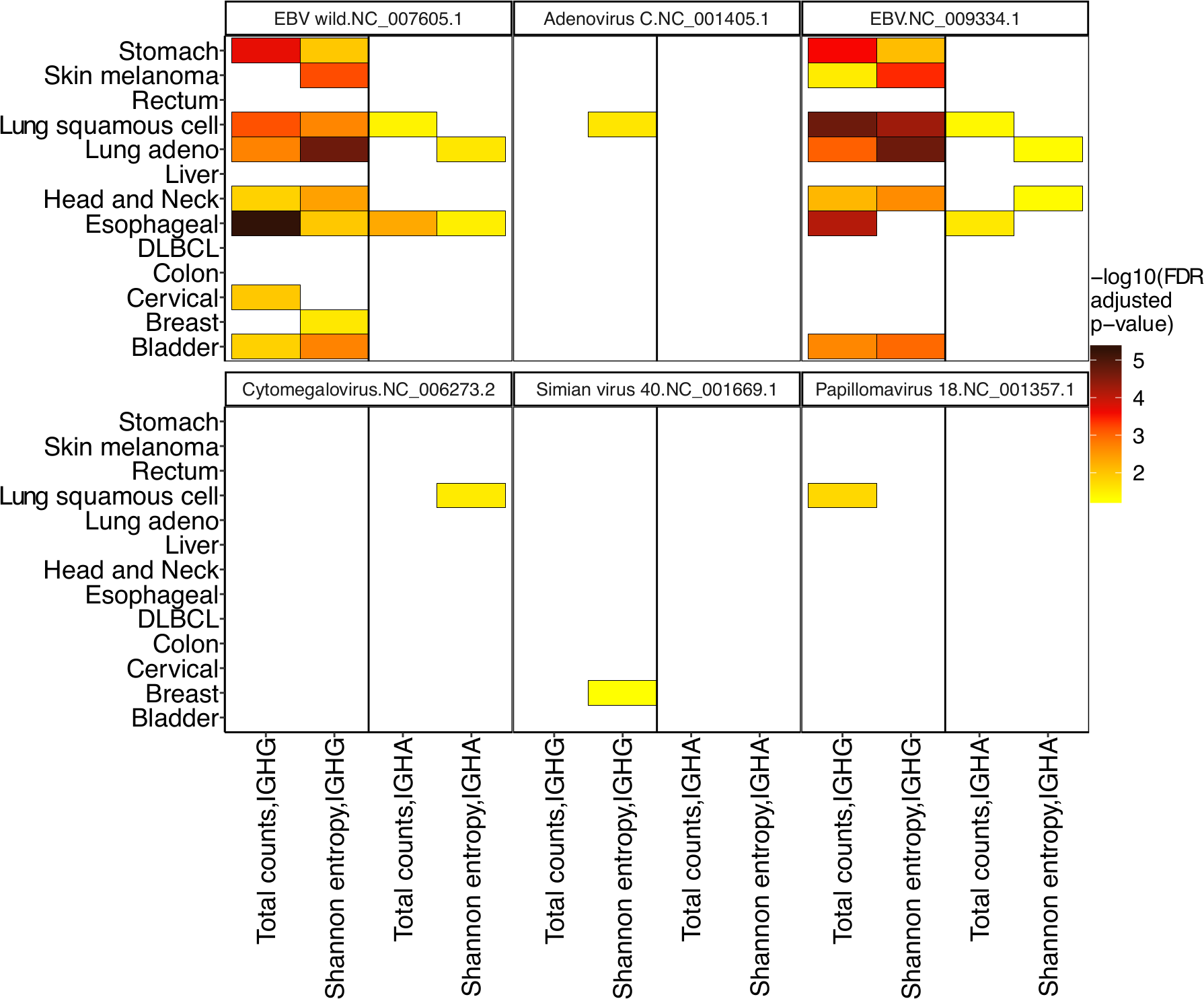

Supplement: FIG S5 [file sys005182263sf5.tif]
